# Context Matters, So How Do We Get Better at Working With Context in Implementation Research and Practice? Comment on "Stakeholder Perspectives of Attributes and Features of Context Relevant to Knowledge Translation in Health Settings: A Multi-country Analysis"

**DOI:** 10.34172/ijhpm.2022.7088

**Published:** 2022-03-02

**Authors:** Gillian Harvey

**Affiliations:** Caring Futures Institute, College of Nursing and Health Sciences, Flinders University, Adelaide, SA, Australia.

**Keywords:** Context, Implementation Practice, Implementation Science, Implementation Practitioners

## Abstract

In the field of implementation research, it is widely recognised that ‘context matters.’ Attempts to implement innovations, research and new knowledge into practice invariably meet contextual challenges at multiple levels during the process of implementation. The paper by Squires and colleagues provides a detailed insight into the many different features and attributes of context. Yet, as this commentary argues, there are significant challenges ahead if we are to apply our growing understanding about context to improve the practice of implementation in everyday healthcare. This will require attention to the practicalities of working with context to achieve successful implementation.

 As the field of implementation science has advanced over the last 15-20 years, it has been increasingly recognised that ‘context matters,’ as it acts as an important mediator of the success or failure of implementation efforts in healthcare.^1^ What works effectively in one setting does not automatically or easily transfer to an alternative setting, even within the same organisation. The detailed examination of the features and attributes of context, undertaken by Janet Squires and colleagues,^2^ provides an important contribution to our understanding of context by methodically unpacking the complexity of the construct and the multiple layers within a seemingly simple term. As the authors point out, implementation researchers and practitioners currently pay attention to context, often aided by determinant implementation frameworks and theories that help to identify contextual barriers and enablers. However, clarity about what specifically matters in terms of context, where it starts and finishes, and how to accurately assess it, is still relatively unknown. Through their detailed and thorough investigation, the authors have produced a more in-depth and nuanced understanding of context that should be beneficial to the implementation research community. They have confirmed existing theoretical and empirical definitions of context and identified 30 new features of context, including a new attribute relating to facility characteristics, that is not represented in previously described implementation theories and frameworks. The resulting taxonomy of 66 features of context, grouped into 16 discrete attributes, expands previous understanding of the construct.

 This deeper understanding of context is an important first step, although there are equally, if not more, demanding challenges ahead. One of these, referred to in the paper, relates to the identification and development of instruments and tools to accurately assess context and support diagnostic assessment before, during and post-implementation. However, it is beyond the accurate description and diagnosis of context that perhaps the most significant challenges remain, in terms of applying our knowledge of context to improve implementation efforts in practice. I will explore these practice-focused challenges by addressing the following three questions:

1. Context matters, but what matters most?
2. How best can we modify and tailor implementation interventions to context?
3. What are the implications of contextual challenges from an implementation research perspective?

## Context Matters, but What Matters Most?

 Give the multitude of factors that potentially influence the translation and implementation of evidence – 66 unique features identified by Squires and colleagues – where should practitioners and researchers start in planning for implementation? Is it reasonable to try and assess the full range of contextual features and attributes, or are some more important than others? From the findings presented in the paper, 2 of the 16 attributes of context, namely culture and resource access, were consistently reported by participating stakeholders, regardless of the country they were working in, their role and level of experience. Acknowledging the potential limitations of the sample interviewed, could this indicate that culture and resource access represent priority issues to consider in the initial planning of an implementation project? Certainly, without a supportive organisational culture and availability of key resources such as time, organisational training and education, and availability of guidelines to inform practice, implementation efforts are reported to be extremely difficult or constrained.^3^ However, it is also important to consider which contextual factors are modifiable and those which are not, at least in the short-term. For example, changing an unsupportive or resistant organisational culture is a significant undertaking and unlikely to be possible for a lone or inexperienced implementation practitioner. Likewise, addressing the issue of lack of time, due to factors such as workload pressures, insufficient staffing or competing priorities, is complex and likely to require long-term strategies and solutions. Thus, whilst we can identify culture and resource access as perhaps the most consistent barriers to implementation, they may not be the ones that are most amenable to modification, an important point and one that links to the next question of tailoring to context.

## Tailoring Implementation to Context

 Within discussions of context in the implementation literature, the importance of tailoring to context is frequently identified as key to the success of implementation.^4^ This implies an ability to assess, identify and respond to contextual barriers and enablers. Yet implementation studies demonstrate how difficult this can be to achieve in practice, because context is typically dynamic and, as noted, may not be easily or immediately modifiable.^5^ Whilst a taxonomy of contextual factors is helpful for assessing likely barriers and enablers of implementation, it does not solve this problem of knowing which contextual factors to respond to, how, when and by whom. For individuals and teams involved in doing implementation – whether they are researchers, practitioners, managers, or a combination – this implies the need for a sophisticated knowledge and skill set, encompassing both relational and technical skills, and a sound understanding of implementation processes.^6^ A systematic review conducted by Albers and colleagues^7^ identifies a range of different role titles assigned to individuals collectively described as ‘implementation support practitioners,’ including, for example, implementation coaches, facilitators and knowledge brokers. The review suggests that despite the different role titles, individuals enacting the roles use a similar set of implementation strategies and share similarities in the skill set required. To build this skill set, the review concludes that:

*“ …. strong professional development pathways are needed to enable staff …. to build, utilize, and offer implementation support skills” *(p. 161).

 Kitson and Harvey^8^ support this idea, proposing a network model of facilitation to develop and mentor implementation facilitators across a continuum of experiential knowledge and skills acquisition from novice to expert. However, whilst knowledge and understanding around roles to support implementation and tailoring implementation interventions to context continues to develop, the fact remains that implementation is notoriously difficult in the most challenging of contexts.^3,9^ So, is there a time when context ‘trumps’ all attempts at implementation, however well the process is supported and facilitated? Or, is it, as Albers and colleagues assert, that we need to increase the repertoire of implementation strategies that are used in order to address a broader range of contextual factors and achieve the level of change agency required.^7^ The detailed taxonomy of context provided by Squires and colleagues^2^ can help to inform this agenda and identify important questions that are in need of further investigation and discussion.

## Implications for Research on Implementation

 Recognising the real-world issues of understanding, responding, and adapting to context also presents a challenge to future thinking about implementation research. Typically, evaluations of implementation interventions employ research designs that seek to control context, or as May and colleagues’ comment^1^:

*“… seek to eliminate contextual confounders, when they represent the normal conditions into which interventions must be integrated if they are to be workable in practice”* (p. 1).

 This raises questions about the way in which implementation research connects to real-world implementation practice, where context is complex, dynamic, and unpredictable. Many trials of implementation interventions include an embedded process evaluation study that provides a more detailed explanation of what did or did not work as planned during implementation and associated reasons why this occurred.^10^ However, these accounts are usually a retrospective description of the contextual factors that influenced implementation, often highlighting substantial variation across different implementation sites with their own specific contextual features. Whilst such accounts help to further build knowledge about implementation, and contextual features that influence implementation success or failure, their retrospective focus limits the ability to effect changes in practice, not least since subsequent implementation efforts will almost certainly encounter a different combination of contextual features. Given the fluid and emergent nature of context, and a desire to accelerate the implementation of innovations and evidence into practice, we need to consider employing a wider range of research designs to evaluate implementation.^11^ This includes methodologies that can account for and work with context as an integral part of the implementation process. For example, realist methodologies help to build an explanatory theory of how context interacts with implementation intervention components to determine whether, how and why the intervention works as planned.^3^ Other approaches to be considered include those that offer the potential to respond to context in real-time including, for example, participatory action research,^12^ integrated knowledge translation,^13^ and developmental evaluation.^14^ This concurs will calls for a ‘plurality of methods’ and viewing context in a more processual (as opposed to deterministic) way, whereby interventions and context evolve in an organic way.^1,15^

## Conclusion

 As others have noted, context presents an important ‘practical problem’ in implementation^1^ and, as such, we need to understand the nature of context and how best to work with it in implementation research and practice. The paper by Squires and colleagues^2^ advances our understanding of the attributes and features of context, but how we use this knowledge will be crucial as we move forward. Implementation science has advanced significantly over recent years, but to move the practice of implementation forward at a similar pace, the science needs to be reflective of reality. This requires a shift in focus and consideration of the practicalities of working *with* context in complex and dynamic environments, developing and mentoring implementation practitioners with the requisite skill set and adopting a broader range of research methodologies to study implementation in context.

## Ethical issues

 Not applicable.

## Competing interests

 Author declares that she has no competing interests.

## Author’s contribution

 GH is the single author of the paper.
